# Supplementary material for: The impact of emotional intelligence on operational effectiveness: The mediating role of organizational citizenship behavior and leadership
Source: PLoS One. 2023 Aug 2;18(8):e0284752. doi: 10.1371/journal.pone.0284752 (PMC10395873; doi:10.1371/journal.pone.0284752)
Supplement: S1 Appendix — (DOCX) [file pone.0284752.s001.docx]

**Appendix A. Measurement Items.**

*Emotional Intelligence*

Self-emotion appraisal (SEA)

I have a good sense of why I have certain feelings most of the time.

I have a good understanding of my own emotions.

I really understand what I feel.

I always know whether or not I am happy.

Others' emotion appraisal (OEA)

I always know my friends' emotions from their behavior.

I am a good observer of others' emotions.

I am sensitive to the feelings and emotions of others.

I have a good understanding of the emotions of people around me.

Use of emotion (UOE)

I always set goals for myself and then try my best to achieve them.

I always tell myself I am a competent person

I am a self-motivated person.

I would always encourage myself to try my best.

Regulation of emotion (ROE)

I am able to control my temper and handle difficulties rationally.

I am quite capable of controlling my own emotions.

I can always calm down quickly when I am very angry.

I have good control of my own emotions.

*Organizational Citizenship Behavior*

Altruism

I help others who have a heavy workload.

I help others who have been absent.

I willingly give my time to help others who have work-related problems.

I help guide new people, even if it's not necessary.

Civic virtue

I make a summary of the changes in the organization.

I attend functions that are not mandatory, but that help the image of the company.

I attend and participate in meetings related to the organization.

Consciousness

I am always punctual.

I don't take extra breaks.

I never take long lunches or breaks.

I obey company rules, regulations, and procedures even when no one is looking.

Sportsmanship

I spend a lot of time complaining about trivial matters.

I constantly talk about wanting to quit my job.

I make problems bigger than they are.

I always focus on what is wrong with my situation, instead of the positive.

Courtesy

I inform my executive or superior before taking important actions.

I consult with my subordinates or others who might be affected by my actions or decisions.

I do not abuse the rights of others.

I take steps to prevent problems with others.

*Leadership*

1. My presence has little effect on the performance of my colleagues.

2. I don't try to change what others do as long as things go well.

3. Colleagues and subordinates are proud to work with me.

4. I place special emphasis on careful problem-solving before acting.

5. I avoid getting involved in the work of others.

6. I do not tell subordinates where I am physically on some occasions.

7. I show that I firmly believe in the saying "if it works, don't fix it".

8. I give others what they want in exchange for their support.

9. I avoid intervening in the work of others, except when the objectives are not achieved.

10. I make sure that there is a strong agreement with others between what I expect them to do and what they can get from me for their efforts.

11. If necessary, the employee can negotiate with me what they will receive in exchange for their work.

12. I let others know that they can achieve what they want if they work as agreed with me.

13. I worry about training (training, educating) those who need it.

14. I focus my attention on the cases in which the expected goals are not achieved.

15. I make people use reasoning and evidence to solve problems.

16. I try to get others what I want in exchange for their cooperation

17. I am willing to instruct them or teach others whenever they need it.

18. I don't try to make changes while things are going well.

19. I give talks to motivate others.

20. I avoid making decisions.

21. I have the respect of colleagues and subordinates.

22. I boost the motivation for the success of others.

23. I try to make others see problems as an opportunity to learn.

24. I try to develop new ways to motivate others.

25. I get others to think about old problems in new ways.

26. I let the others continue doing their work as they have always done if I do not think it is necessary to introduce some change.

27. I am hard to find when a problem arises.

28. I encourage the use of intelligence to overcome obstacles.

29. I ask others to support their opinions with solid arguments.

30. I give them new ways to approach problems that used to be perplexing.

31. I avoid telling others how to do things.

32. I am likely to be absent when needed.

33. Others have full confidence in me.

34. They trust my ability to overcome any obstacle.

*Operational effectiveness*

Quality

This organization seeks to deliver value-adding products or services of improved quality.

Our organization regards quality as the most important competitive priority.

Our organization has demonstrated a commitment to quality.

This organization measures what it does and seeks to improve business processes.

Our organization is seeking opportunities to bridge the gap between organizational capabilities and customer demand.

The organization is looking for a consistent provision of products and services that satisfy customers.

Speed

This organization ensures it delivers services/products on time.

This organization is able to shorten the time between the service or product request and the service or product delivery.

This organization is able to deliver a product or service at the frequency and time expected by customers/users.

In this organization, products/services are delivered on time.

Cost

Offering competitive pricing is important to our organization.

The organization is seeking to eliminate waste through efficient processes such as purchasing, production, and staff performance.

Our organization is cost effective.

It is important to make communication cost efficient.

It is important to minimize travel costs.

It is important to keep the cost of services down.

Flexibility

Our organization has the ability to adjust to changes in order to respond to customer/user demands.

Our organization can easily change the procedures required for a service.

The organization could offer better-customized services/products.

Reliability

The organization's processes consistently perform as expected.

Our products/services meet customer requirements.

Our organization is concerned that the product/service is presenting errors.

The services offered are accurate.

Our organization is consistently delivering or providing products/services that the customer can rely on.
