# Supplementary material for: The impact of emotional intelligence on operational effectiveness: The mediating role of organizational citizenship behavior and leadership
Source: PLoS One. 2023 Aug 2;18(8):e0284752. doi: 10.1371/journal.pone.0284752 (PMC10395873; doi:10.1371/journal.pone.0284752)
Supplement: Attachment — Submitted filename: Answer to Reviewers.docx [file pone.0284752.s002.docx]

Answer to Reviewers

| Request | Action |
| --- | --- |
| Please remove your figures from within your manuscript file, leaving only the individual TIFF/EPS image files.  These will be automatically included in the reviewer’s PDF. | The file with the figures was removed from the submission. |
| Please ensure that you include a title page within your main document. You should list all authors and all affiliations as per our author instructions and clearly indicate the corresponding author. | A title page within the main document was included with a list of the authors and the corresponding author. |
| Please provide additional details regarding participant consent. In the ethics statement in the Methods and online submission information, please ensure that you have specified what type you obtained (for instance, written or verbal, and if verbal, how it was documented and witnessed). If your study included minors, state whether you obtained consent from parents or guardians. If the need for consent was waived by the ethics committee, please include this information. | In the document we included the following statement:  To avoid the social desirability bias, the ethics committee at the Colegio de Estudios Superiores de Administración (CESA) in Colombia reviewed the questionnaire to ensure that questions were neutral, unbiased, and non-threatening. Additionally, before filling out the questionnaire, the respondents were informed that all information provided would be treated in the strictest confidence, that the responses would be aggregated and used for research purposes, and that by completing this survey, they were giving their consent to such use, and that they might withdraw at any time. The researchers followed ethical procedures during this research project by adhering to ethical standards.    Additionally, we included the following statement in the submission page in the ethical statement section:  The study was approved by the Ethics Committee at Colegio de Estudios Superiores de Administración - CESA. Approval number 006 date 25-04-2022  Cometee Members: Dr. Edgardo Cayón (Research Director); Dr. Rodrigo Zarate (Full-time Professor); Dr. Javier Cadena (Full-time Professor); Dr. Ricardo Santa (Full-time Professor)  Date: April 25th, 2022  The IRB Clearance document is attached under the "other" in this submission  The questionnaire respondents have the following information before filling up the questionnaire:  CONFIDENTIALITY ASSURANCE  • All information provided will be treated in strictest confidence.  • The responses will be aggregated and used to examine research issues.  • At no stage will any of the information be divulged to third parties in their disaggregated form.  • You may withdraw at any time.  • By completing this survey you are giving your consent.  Ethical procedures were adopted by the researchers when building this research project. |
